# St. John’s wort extract and hyperforin protect rat and human pancreatic islets against cytokine toxicity

**DOI:** 10.1007/s00592-013-0518-2

**Published:** 2013-10-12

**Authors:** Michela Novelli, Pascale Beffy, Marta Menegazzi, Vincenzo De Tata, Luisa Martino, Anna Sgarbossa, Svetlana Porozov, Anna Pippa, Matilde Masini, Piero Marchetti, Pellegrino Masiello

**Affiliations:** 1 Department of Translational Research and New Technologies in Medicine and Surgery, University of Pisa, Via Roma 55, 56126 Pisa, Italy; 2 Institute of Clinical Physiology, CNR, Via Moruzzi 1, 56124 Pisa, Italy; 3 Department of Life and Reproduction Sciences, Biochemistry Section, University of Verona, Strada Le Grazie 8, 37134 Verona, Italy; 4 Scuola Superiore Sant’Anna, Pisa, Italy; 5 Department of Clinical and Experimental Medicine, University of Pisa, Via Roma 67, 56126 Pisa, Italy

**Keywords:** Type 1 diabetes, Cytokines, Human pancreatic islets, Beta-cell death, St. John’s wort, Hyperforin

## Abstract

The extract of *Hypericum perforatum* (St. John’s wort, SJW) and its component hyperforin (HPF) were previously shown to inhibit cytokine-induced activation of signal transducer and activator of transcription-1 and nuclear factor κB and prevent apoptosis in a cultured β-cell line. Objective of this study was to assess the protection exerted by SJW and HPF on isolated rat and human islets exposed to cytokines in vitro. Functional, ultrastructural, biomolecular and cell death evaluation studies were performed. In both rat and human islets, SJW and HPF counteracted cytokine-induced functional impairment and down-regulated mRNA expression of pro-inflammatory target genes, such as iNOS, CXCL9, CXCL10, COX2. Cytokine-induced NO production from cultured islets, evaluated by nitrites measurement in the medium, was significantly reduced in the presence of the vegetal compounds. Noteworthy, the increase in apoptosis and necrosis following 48-h exposure to cytokines was fully prevented by SJW and partially by HPF. Ultrastructural morphometric analysis in human islets exposed to cytokines for 20 h showed that SJW or HPF avoided early β-cell damage (e.g., mitochondrial alterations and loss of insulin granules). In conclusion, SJW compounds protect rat and human islets against cytokine effects by counteracting key mechanisms of cytokine-mediated β-cell injury and represent promising pharmacological tools for prevention or limitation of β-cell dysfunction and loss in type 1 diabetes.

## Introduction

In type 1 diabetes, destruction of pancreatic β-cells is believed to be mainly carried out by cytokines released from infiltrating lymphocytes and macrophages abnormally activated by an autoimmune response against various self-β-cell antigens in genetically susceptible subjects [1–3]. Both apoptosis and necrosis have been reported to be responsible for cell death in pancreatic β-cells exposed to immune and inflammatory cytokines [4–6] that can induce the expression of various pro-inflammatory and pro-apoptotic genes, essentially through the concomitant activation of the signal transducer and activator of transcription-1 (STAT-1) and the nuclear factor kB (NF-kB). Of these transcription factors, the former is activated by Th-1 lymphocyte-derived IFN-γ and the latter by either IL-1β or TNF-α produced by effector macrophages during the autoimmune response triggered in the pancreatic islet environment. STAT-1 proteins, which are present in an inactive monomeric form in the cytosol, when phosphorylated by the IFN receptor-associated Janus tyrosine kynases 1 and 2 (JAK 1-2), form dimers and translocate to the nucleus where they bind to γ-activated sequence elements in the regulatory regions of numerous pro-inflammatory and pro-apoptotic genes [7–10]. Similarly, NF-kB remains inactive in the cytoplasm under basal conditions, bound to the inhibitory molecule IkB. Following stimulation by IL-1β and/or TNF-α, IkB is phosphorylated and degraded in the proteasome, allowing NF-kB to undergo phosphorylation in its p65 subunit and subsequently move to the nucleus [11] to induce transcription of many genes containing NF-kB sites in their promoter regions, including iNOS, chemokines, adhesion molecules and other inflammatory factors. In most cases, the concurrent activation of STAT-1 and NF-kB pathways by a cytokine mixture is required for the full induction of target genes and consequent severe β-cell damage [12–14]. Thus, the regulation of both these signalling pathways appears crucial to achieve an effective protection of β-cells against the deleterious effects of cytokines. Much interest has been recently raised on compounds of vegetal origin (mainly polyphenols and flavonoids), which have been shown to exert inhibitory effects on cytokine signalling in β-cells [15–18]. In view of future clinical applications, these non-peptidyl compounds are particularly attractive, as they are non-immunogenic, available for chronic oral administration, and usually devoid of major side effects [19] .

We have recently focused our attention on the promising anti-inflammatory properties of the extract of *Hypericum perforatum* (St. John’s wort, SJW), a herbaceous plant diffused in Mediterranean area, used as anti-depressant due to its ability to inhibit neuronal reuptake of amine neurotransmitters and its favourable tolerability profile [20, 21]. Based on the demonstration of a potent inhibitory effect of SJW extract on IFNγ-induced STAT-1 activation in tumoural cell lines [22], we observed that in the well-differentiated pancreatic β-cell line INS-1E exposed to a cytokine mixture, SJW extract and its component hyperforin (HPF) were able to exert a similar inhibitory effect not only on STAT-1 but also on NF-kB activation, which resulted in prevention of cytokine-induced β-cell damage [23]. Taking into account that prevention of cytokine-induced STAT-1 and NF-kB activation by these vegetal compounds was also observed in isolated rat and human pancreatic islets [23], in this work we aimed at assessing whether SJW extract and HPF would effectively protect rat and human islets against cytokine-induced beta-cell dysfunction, inflammatory response and cell death. The confirmation of the effectiveness of SJW components in counteracting cytokine damaging effects in a physiological environment such as whole islet tissue appears an essential step in view of potential preventive or therapeutic interventions based on these vegetal compounds, not only in pre- and early type 1 diabetes to delay or attenuate autoimmune beta-cell destruction, but also during the post-transplantation period to help preserving integrity and function of transplanted islets in severely diabetic patients.

## Materials and methods

### Reagents

Rat and human IFN-γ, IL-β and TNF-α were purchased from PetroTech Inc. (London, England). A standardized, HPLC-titrated hydro-alcoholic extract of SJW, containing 4.1 % HPF, was obtained from Indena (Milan, Italy) and dissolved in DMSO at high concentration just before proper dilution (at least 1:200) in the culture medium. HPF was purchased from Sigma (St. Louis, MO, USA) as a 250 μg/ml solution in methanol (465 μM) and used after suitable dilutions in the culture medium.

### Isolation and incubation of rat pancreatic islets

Pancreatic islets were isolated from male Sprague–Dawley rats of 200–250 g b.w. (Harlan Italy) as described elsewhere [24]. The experimental protocol followed the Principles of Laboratory Animal Care (US NH publication No. 83-85, revised 1985) and was approved by the Ethical Committee of the University of Pisa. Isolated islets were resuspended in RPMI culture medium (containing 11 mM glucose, antibiotics and 10 % adult bovine serum) and cultured at 37 °C in 5 % CO_2_ for 24 h, before use.

### Isolation and incubation of human pancreatic islets

Human pancreatic islets were isolated as previously described [25] from pancreata of non-diabetic multi-organ donors with the approval of the Ethics Committee of the University of Pisa, after an informed consent was obtained in written form from family members. Isolated islets were resuspended in M199 culture medium (containing 5.5 mM glucose, antibiotics and 10 % adult bovine serum), cultured at 37 °C in 5 % CO_2_ and studied within 3–4 days from isolation.

### Functional studies

Isolated rat or human islets were incubated for 20 h in 1 ml of fresh RPMI or M199 medium, respectively, containing 10 % FCS and a cytokine mixture (rat or human IFN-γ 400 U/ml + IL-1β 50 U/ml + TNF-α 200 U/ml), with or without SJW (200 μg/ml) or HPF (2 μM). Then, batches of 10–15 islets, after 30-min pre-incubation, were incubated for 1 h in Krebs-Ringer-Bicarbonate-Hepes buffer containing 2.8 or 16.7 mM glucose. The insulin released and that extracted from the islets by acidified ethanol was measured by radioimmunoassay, as described [24].

### Real-time PCR

Batches of 250 rat or human islets were incubated in 0.8 ml of RPMI or M199 medium, respectively, containing 5 % FCS and SJW or HPF for 45 min. Then, a cytokine mixture (IFN-γ 1,000 U/ml + IL-1β 100 U/ml + TNF-α 1,000 U/ml) was added for subsequent 20-h incubation. Then, the islets were washed, pelleted and stored at −80 °C to be later analysed by real-time PCR. After extraction in guanidine-thiocyanate buffer, total islet RNA was purified by RNeasy Mini spin column (QIAGEN) and quantified with NanoDrop 1000 spectrophotometer. RNA was reverse transcribed into cDNA, using SuperScript^®^ VILO cDNA Synthesis Kit (Invitrogen). The real-time PCR amplification reaction was done in triplicates, using the QuantiTect SYBR Green PCR kit (QIAGEN) and the Rotor-Gene™ 6000 (Corbett Research), as previously described [26]. The specificity of the amplification was assessed by the occurrence of a single peak in the melting curve; the efficiency of the reaction was in the range 1.75–1.90. Results of real-time PCRs were analysed by the Pfaffl method and normalized to cytokine-exposed samples. Expression values were corrected for the housekeeping gene 18S that remained stable in the various experimental conditions, likewise glyceraldehydes-3-phosphate dehydrogenase and β-actin which were also tested as control genes. The following primers (QuantiTect Primer Assay, QIAGEN) were used for human and rat iNOS, CXCL9, CXCL10, COX2, respectively: Hs_NOS2_1_SG, Rn_Nos2_1_SG; Hs_CXCL9_1_SG, Rn_Cxcl9_1_SG; Hs_CXCL10_1_SG, Rn_Cxcl10_1_SG; Hs_PTGS2_1_SG, Rn_Ptgs2_1_SG; and Hs_RRN18S_1_SG for 18S (reference gene).

### Nitric oxide measurement

NO was measured as accumulated nitrites in the medium after 48 h of exposure to a cytokine mixture, with/without SJW extract or HPF, by mixing equal volumes of medium and Griess reagent (0.1 % naphthylethene diamine hydrochloride (Sigma) in water and 1 % sulphanilamide (Sigma) in 5 % H_3_PO_4_). The absorbance was measured at 540 nm and nitrite calculated from a NaNO_2_ standard curve.

### Evaluation of apoptosis and necrosis

Batches of 10–15 rat or human islets were incubated in 1 ml of RPMI or M199 medium, respectively, containing 10 % FCS and various concentrations of SJW extract (50, 100 and 200 μg/ml) or HPF (1 and 2 μM) for 30 min. Then, a cytokine mixture (rat or human IFN-γ 1,000 U/ml + IL-1β 100 U/ml + TNF-α 1,000 U/ml) was added for subsequent 48-h incubation. At the end of this period, culture medium was used for necrosis and the cell lysate for apoptosis determinations, simultaneously assessed following the instructions of the Cell Death Detection ELISAplus kit (Roche, Basel, Switzerland), based on the measurement of the amount of both released and intracellular (cytoplasmic) histone-associated DNA fragments.

### Ultrastructural and morphometric analysis

After 20-h exposure to a cytokine mixture (IFN-γ 1,000 U/ml + IL-1β 100 U/ml + TNF-α 1,000 U/ml), with/without SJW extract or HPF, isolated human islets were fixed in 2.5 % glutaraldehyde in 0.1 M phosphate buffer (pH 7.3) for 2 h at 4 °C, washed in 0.1 M phosphate buffer (pH 7.4), post-fixed in 0.1 % osmium tetroxide in the same buffer and dehydrated in a graded series of ethanol. Then, the islets were transferred to propylene oxide and embedded in PolyBed 812 (Polyscience Inc., Warrington, Pa, USA). Ultrathin sections were stained with uranyl acetate and lead citrate and observed under a Zeiss 902 transmission electron microscope. Morphometric analysis was independently performed by two researchers (M.M. and L.M) using stereological techniques, as described [27].

### Calculations and statistical analysis

Data are presented as mean ± SEM. Differences between means were tested for statistical significance using factorial analysis of variance followed by Tukey–Kramer multiple comparisons test as the post hoc test (InStat 3, GraphPad software).

## Results

### SJW and HPF prevent cytokine-elicited impairment in glucose-stimulated insulin secretion from rat and human islets

**Fig. 1:**
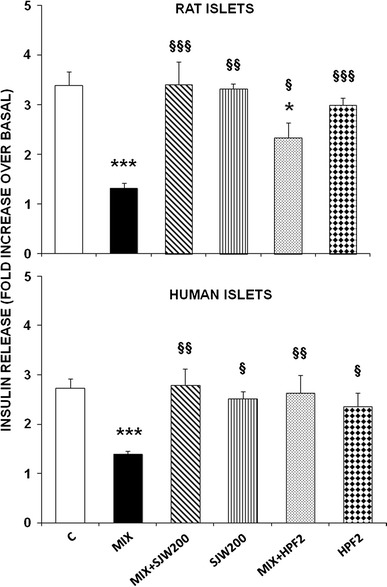
Effects of SJW and HPF on cytokine-induced impairment in glucose-stimulated insulin release in rat and human islets. Isolated rat or human islets (batches of 20 islets) were incubated for 20 h in RPMI or M199 medium containing a cytokine mixture (MIX), in the absence or presence of either SJW (200 μg/ml) or HPF (2 μM). Then, after removal of the medium, the cells were washed and incubated for 1 h in KRB/Hepes buffer with 2.8 or 16.7 mM glucose. Glucose-stimulated insulin release was expressed as fold increase over basal. Mean ± SEM of 10–12 observations from 3 separate experiments. *p < 0.05, ***p < 0.001 versus control islets; ^§^ *p* < 0.05, ^§§^ *p* < 0.01, ^§§§^ *p* < 0.001 versus islets exposed to MIX

Following 20-h exposure of rat or human islets to cytokines, glucose-stimulated insulin secretion was measured after 1-h incubation in KRB/Hepes buffer (Fig. 1). At 16.7 mM glucose, insulin secretion, normalized to islet total insulin content, increased about threefold over basal in both rat and human control islets, but was markedly impaired in islets previously exposed to cytokines. However, when either SJW (200 μg/ml) or HPF (2 μM) was present during cytokine exposure, glucose-stimulated insulin secretion was restored to normal values, with the partial exception of rat islets in which HPF treatment was not fully protective. Vegetal compounds per se did not significantly modify glucose-stimulated insulin release.

### SJW and HPF down-regulate cytokine-induced expression of pro-inflammatory genes

**Fig. 2:**
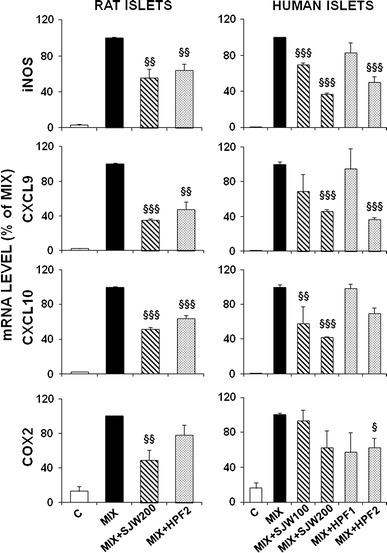
Effects of SJW extract and HPF on cytokine-induced expression of pro-inflammatory target genes in rat and human islets. After 20-h exposure to a cytokine mixture (MIX) in RPMI (rat islets) or M199 (human islets) medium, with or without SJW (100**–**200 μg/ml) or HPF (1**–**2 μM), total islet RNA was extracted and analysed by real-time PCR. Data are mean ± SEM of 3–4 independent experiments and are expressed as percentage of cytokine-induced gene expression, assumed as 100 %, after correction with the housekeeping gene 18S. ^§^ *p* < 0.05, ^§§^ *p* < 0.01, ^§§§^ *p* < 0.001 versus MIX

The mRNA expression of relevant pro-inflammatory genes (iNOS, CXCL9, CXCL10, COX2), as evaluated by real-time PCR, was markedly induced by 20-h cytokine treatment in both rat and human islets (Fig. 2). In rat islets, SJW extract (200 μg/ml) and HPF (2 μM) were able to significantly hamper this induction (in most cases, *p* < 0.01 at least), being the inhibitory effect of SJW slightly stronger than that of HPF. In human islets, the enhanced expression of the pro-inflammatory genes was also down-regulated in the presence of either SJW (100–200 μg/ml) or HPF (1–2 μM) in a dose-dependent manner, taking, however, into account that the low dose of HPF was in most cases ineffective.

### SJW and HPF inhibit cytokine-induced nitrites production

**Fig. 3:**
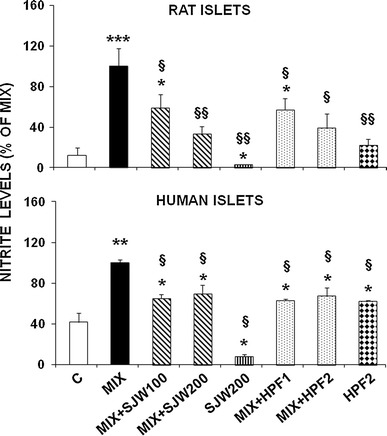
Effects of SJW and HPF on cytokine-induced nitrite accumulation in isolated rat and human islets. Isolated rat or human islets were exposed for 48 h to a cytokine mixture (MIX) and various doses of SJW (100**–**200 μg/ml) or HPF (1**–**2 μM). Nitrites accumulated in the medium were measured using the Griess method. Data are presented as mean ± SEM of 5 and 3 observations in rat and human islets, respectively, after normalization for the number of islets. *p < 0.05, **p < 0.01, ***p < 0.001 versus controls; ^§^ *p* < 0.05, ^§§^ *p* < 0.01 versus MIX

As shown in Fig. 3, in rat islets exposed for 48 h to cytokines, the remarkable accumulation of nitrites into medium, a valuable index of NO production, was significantly and progressively abated by increasing concentrations of both SJW and HPF (*p* < 0.05 or *p* < 0.01 according to the dose). In human islets, similar results were obtained, being the vegetal compounds able to significantly reduce (*p* < 0.05) the cytokine-induced increment of nitrites in the medium, although no dose-dependent effect was observed.

### SJW and HPF protect rat and human islets against cytokine-induced cell death

**Fig. 4:**
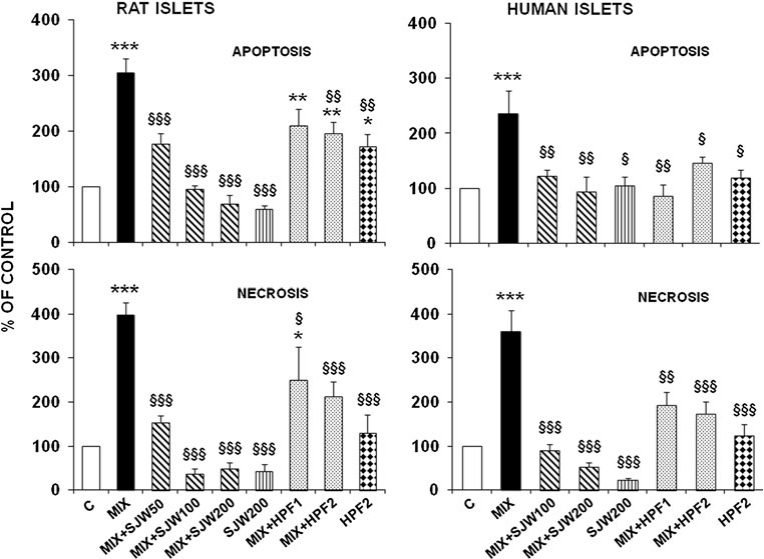
Protective effects of SJW and HPF on cytokine-induced apoptosis and necrosis in isolated rat and human islets. Isolated rat or human islets were exposed for 48 h to a cytokine mixture (MIX) and various doses of SJW (50**–**200 μg/ml) or HPF (1**–**2 μM). Apoptosis and necrosis were determined using the Cell Death detection ELISA^PLUS^ kit (Roche). Data are expressed as percentage of controls and are mean ± SEM of 5–8 independent observations. *p < 0.05, **p < 0.01, ***p < 0.001 versus controls; ^§^ *p* < 0.05, ^§§^ *p* < 0.01, ^§§§^ *p* < 0.001 versus MIX

A 48-h exposure of rat isolated islets to a cytokine mixture resulted in a fourfold increase in necrosis and a threefold increase in apoptosis. Cytokine-induced apoptosis and necrosis were dose-dependently prevented by SJW extract in the range 50–200 μg/ml, with necrosis being inhibited even below control values at higher SJW concentrations, while the preventive effect of 1 or 2 μM HPF was only partial (Fig. 4, rat islets). The protective effects of SJW and HPF were substantially confirmed in human islets, with some differences in the extent of the protection (Fig. 4, human islets). In fact, cytokine-induced apoptosis was fully and similarly prevented by SJW (100–200 μg/ml) and HPF (1–2 μM), while the anti-necrotic effect of SJW was stronger than that exerted by HPF.

### SJW and HPF prevent early damage in human islets exposed to cytokines, as assessed by ultrastructural and morphometric analysis

**Fig. 5:**
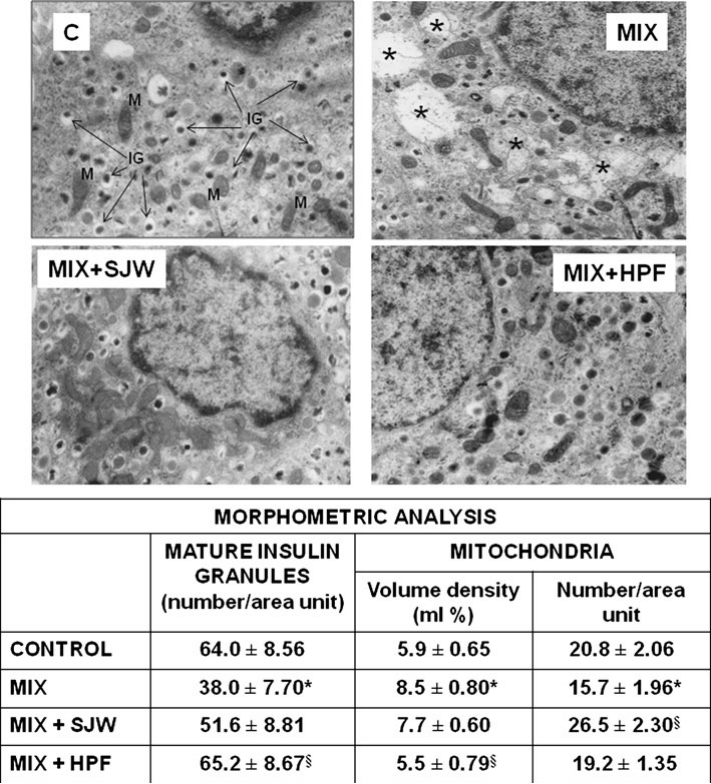
Protective effects of SJW and HPF on cytokine-induced ultrastructural alterations in β-cells of isolated human islets, as assessed by morphometric analysis. Representative images of β-cells from isolated human islets after 20-h incubation in M199 medium containing a cytokine mixture (MIX), with or without SJW extract (100 μg/ml) or HPF (2 μM). Magnification: ×16,000. In the control (*C*) panel, M indicates normal mitochondria, IG with black arrows points at mature insulin granules (with a typical dense core surrounded by a large white halo). A few immature insulin granules (with a less dense core and thin halo) are also visible. In the MIX panel, *asterisks* mark damaged mitochondria. In the MIX + SJW panel, a large cluster of normal mitochondria appears. Morphometric analysis was performed as detailed elsewhere [27], being the number of insulin granules and mitochondria referred to a real area unit of 220 μm^2^ and the volume density of mitochondria expressed in ml/100 ml of tissue (ml %). Given parameters are expressed as mean ± SEM of at least 10 different measurements. *p < 0.05 versus controls; ^§^ *p* < 0.05 versus MIX

Ultrastructural images of human islets exposed to cytokines for 20 h (Fig. 5) revealed abnormalities in subcellular organelles in β-cells, in particular paucity of insulin granules and mitochondrial swelling with rupture and disappearance of cristae. These early signs of β-cell damage did not occur in the presence of either SJW or HPF, as confirmed by morphometric analysis of micrographs (bottom of Fig. 5). Indeed, morphometric measurements clearly indicate that the vegetal compounds prevent cytokine-induced reduction in the number of β-cell granules and mitochondria and avoid increase in mitochondrial volume density. In the presence of SJW, clusters of normal mitochondria were often observed, probably contributing to the trend to increased number and volume density of these organelles with respect to control islets, as revealed by the morphometric measurements.

## Discussion

There is general consensus that immune and inflammatory cytokines released by mononuclear cells infiltrating pancreatic islets during an autoimmune attack are crucial mediators in the destruction of beta cells in type 1 diabetes [1–3]. There are also increasing evidences that β-cell death elicited by systemic or localized overproduction of inflammatory cytokines in obesity [28], hyperglycaemic conditions [29] or during the post-transplantation period [30] may strongly contribute to the β-cell loss occurring in type 2 diabetes [31] or in islet graft failure [30]. It should be reminded that, at least in vitro conditions, β-cell death is usually induced by combination of different pro-inflammatory cytokines (IFN-γ plus IL-β and/or TNF-α) and not by each of them alone [4, 13, 14], thereby probably requiring concomitant activation of both JAK/STAT and NF-kB signalling and transcriptional pathways. Various reports have documented that either NF-kB molecular blockade [32, 33] or STAT-1 genetically engineered deficiency [34] can each protect pancreatic β-cells against cytokine-induced cell death. In our laboratory, we showed that in the β-cell line INS-1E, non-peptidyl compounds of vegetal origin, namely SJW extract and the phloroglucinol hyperforin, one of its main components, acted as potent inhibitors of both STAT-1 and NF-kB cytokine-induced activation and prevented β-cell dysfunction, iNOS expression and apoptosis [23]. Furthermore, we showed that these compounds strongly inhibited STAT-1 and NF-kB activation also in isolated rat and human islets exposed to a cytokine mixture [23]. On this basis, to support and extend the physiological relevance of the results obtained from the β-cell line studies, we explored the protective effects of SJW extract and HPF using pancreatic islets isolated from both rats and humans and exposed to cytokine mixtures for various time periods, according to the parameters to be measured.

Actually, this is the first demonstration that SJW compounds effectively counteract cytokine effects in human islets, as they prevent β-cell dysfunction and early damage, pro-inflammatory response and β-cell death. Other natural compounds, mostly polyphenols and flavonoids, were shown to exert some protective effects in β-cell lines or rodent β-cells [15–18], but they have never been tested in human samples, with the exception of a plant steroidal lactone (Withaferin A), that was very recently reported to inhibit cytokine-induced damage in cultured human islets [35].

Despite a certain unavoidable variability in islets preparations and the limited availability of human islets in particular, it should be underlined that the findings of our study are fairly consistent, with regard to both the similarity of the results between rat and human islets and the effects of the protection exerted by the vegetal compounds on the measured parameters. In particular, glucose-stimulated insulin release from isolated islets, impaired upon cytokine treatment, was restored in the presence of SJW or HPF, confirming results obtained in INS-1 cell line [23]. Expression of a number of pro-inflammatory genes, including iNOS, was markedly enhanced during cytokine exposure and was in most cases significantly down-regulated in presence of SJW extract or HPF, in both rat and human islets. Among the genes examined, COX-2 was the less sensitive to the protective action of the vegetal compounds, as reduction in its expression did not always achieve statistical significance. These data fit with the significant reduction in NO-dependent nitrite accumulation in the medium of rat and human islets exposed to cytokines and protected with SJW or HPF.

A major finding of our investigation is that SJW extract was able to fully prevent cytokine-induced apoptosis and necrosis in both rat and human islets. This protective effect was increasing with increasing dose of the extract (in the range 50–200 μg/ml), so that necrosis was reduced even below the levels of controls. HPF exerted similar anti-apoptotic and slightly smaller anti-necrotic activity in human islets, but was only partially effective in rat islets. We cannot exclude that for rat islets, the supra-physiological glucose concentration in the medium (11 mM) might have augmented the cytokine-induced alterations, but we consider this unlikely, as we could observe no toxic effect in rat islets cultured at glucose concentrations up to 25 mM [36].

Taking into account that the effectiveness of SJW extract is almost entirely dependent on its content in HPF (4.1 % in the HPLC-titrated extract used) [23] and that pure HPF exerted a stronger inhibitory effect on islet STAT-1 and NF-kB activation than HPF contained in equimolar concentrations in the SJW extract [23], we suppose that the stronger protection exerted by SJW extract with respect to HPF alone may be related to the antioxidant or stabilizing properties of other components of the extract that might help preserving HPF activity in long-term in vitro incubations. Further experiments are required to confirm such an hypothesis.

We were also able to evaluate by ultrastructural and morphometric analysis the early β-cell damage caused by cytokines, i.e., loss of insulin granules and mitochondrial changes. With regard to mitochondrial volume density, a parameter that depends on both mitochondrial number and size, it should be noticed that its significant increase upon cytokine treatment, despite reduction in mitochondrial number, implies a conspicuous dilation of these organelles and suggests a possible link with their permeabilization and consequent β-cell dysfunction and triggering of the intrinsic apoptotic pathway [37]. Actually, both SJW extract and HPF fully preserved β-cell granulation and mitochondrial integrity in islets exposed to cytokines. Interestingly, the presence of SJW was associated with an increase in mitochondrial number with respect not only to islets exposed to cytokines but also to control islets, resulting in a mitochondrial volume density above control values. It is worth noticing that no apoptotic or necrotic features could be observed at ultrastructural level in islets exposed for 20 h to cytokines.

In conclusion, our results provide for the first time evidence that SJW extract and its component HPF effectively protect rat and human islets against cytokine-induced β-cell dysfunction, inflammatory response and death, by counteracting key mechanisms of cytokine signalling and transduction pathways, such as STAT-1 and NF-kB, as previously documented [23]. Studies are in progress in our laboratory to establish which steps in cytokine-mediated signalling cascades in β-cells are specifically targeted by these vegetal compounds. Yet, the protective effects obtained in the physiological context of rodent and human islets represent a promising achievement in view of innovative pharmacological intervention for prevention or limitation of β-cell dysfunction and loss in type 1 diabetes and improvement of islet transplant outcome.
